# Si-Based Materials for Thermoelectric Applications

**DOI:** 10.3390/ma12121943

**Published:** 2019-06-17

**Authors:** Sora-at Tanusilp, Ken Kurosaki

**Affiliations:** 1 Graduate School of Engineering, Osaka University, 2-1 Yamadaoka, Suita, Osaka 565-0871, Japan; 2 Institute for Integrated Radiation and Nuclear Science, Kyoto University, 2 Asashiro-Nishi, Kumatori-cho, Sennan-gun, Osaka 590-0494, Japan; 3 Research Institute of Nuclear Engineering, University of Fukui, 1-3-33 Kanawa-cho, Tsuruga, Fukui 914-0055, Japan

**Keywords:** thermoelectric, Si, nanocomposite, nanostructuring, melt spinning, YbSi_2_

## Abstract

Si-based thermoelectric materials have attracted attention in recent decades with their advantages of low toxicity, low production costs, and high stability. Here, we report recent achievements on the synthesis and characterization of Si-based thermoelectric materials. In the first part, we show that bulk Si synthesized through a natural nanostructuring method exhibits an exceptionally high thermoelectric figure of merit *zT* value of 0.6 at 1050 K. In the second part, we show the synthesis and characterization of nanocomposites of Si and metal silicides including CrSi_2_, CoSi_2_, TiSi_2_, and VSi_2_. These are synthesized by the rapid-solidification melt-spinning (MS) technique. Through MS, we confirm that silicide precipitates are dispersed homogenously in the Si matrix with desired nanoscale sizes. In the final part, we show a promising new metal silicide of YbSi_2_ for thermoelectrics, which exhibits an exceptionally high power factor at room temperature.

## 1. Introduction

Thermoelectric (TE) devices have received attention for generating power from waste heat because they can directly convert temperature gradients into electricity. The efficiency of a TE device is determined by the temperature gradient across the device as well as the properties of the TE material, as characterized by the dimensionless figure of merit *zT* = *S*^2^*σTκ*^−^^1^ [1,2], where *S* is the Seebeck coefficient, *σ* is the electrical conductivity, *T* is the absolute temperature, and *κ* is the total thermal conductivity (*κ* = *κ*_lat_ + *κ*_el_, where *κ*_lat_ and *κ*_el_ are the lattice and electronic contributions, respectively).

State-of-the-art high-*zT* materials such as Bi_2_Te_3_ [3] and PbTe [4] contain highly toxic and/or rare elements, which can limit their applicability. In order to expand the practical use of TE power generation in commercial markets, inexpensive and non-toxic TE materials are required. Si-based materials are non-toxic, inexpensive, and Earth-abundant, and therefore extraordinarily advantageous for commercial utilization in TE power generation. For decades, various Si-based TE materials such as Si–Ge alloys and Mg_2_Si have been developed. Si–Ge alloys are well-known as one of the best materials for TE power generation at high temperature [5,6], with a maximum *zT* value of around unity at 1100 K [6]. Similarly, it is considered that some metal silicides have potential to be a good TE material. One of the best metal silicides is Mg_2_Si. It has been demonstrated that Mg_2_(Si,Sn) shows *n*-type characteristics with a *zT* value of above unity at 700 K [7,8]. For *p*-type metal-silicide TE materials, higher manganese silicides have been developed, in which the maximum *zT* value of 0.7 at 800 K has been confirmed [9,10]. Although bulk Si exhibits good electrical properties, its *κ*_lat_ is very high (>140 W·m^−1^·K^−1^ at 300 K for a non-doped single crystal [11,12]), yielding a low *zT* value of ~0.02 at maximum at 300 K [12]. Therefore, recent research on Si-based TE materials has focused on methods to reduce *κ*_lat_. In recent years, significant improvements in *zT* in various traditional TE materials have been achieved, particularly via considerable methods for the minimization of *κ*_lat_ including alloy scattering [13,14], rattling [15,16], lone-pair electrons [17,18], and nanostructuring [19,20,21,22,23,24,25,26]. Nanostructuring permits *κ*_lat_ reduction while maintaining high electrical properties [19,20,21,22,23,24,25,26]. In addition to Si, many metal silicides are considered advanced TE materials because of their low toxicities and high chemical stabilities.

In this paper, we report our recent accomplishments in the synthesis and characterization of Si-based TE materials. First, we describe a natural nanostructuring method that produces nanoscale precipitates in the Si matrix [26]. The nanostructured bulk Si achieved by this method shows a greatly decreased *κ*_lat_ but no significant changes in the electron transport properties, yielding an exceptionally high *zT* value of ~0.6 at 1050 K [26]. Next, a rapid-solidification melt-spinning (MS) technique is described in the synthesis of Si–metal silicide nanocomposites [26]. Unlike traditional ball-milling methods, the MS technique allows for natural formation of nanoscale precipitates, which can prevent some of the disadvantages experienced by TE materials, such as contamination and oxidation. The MS technique has been applied to make nanostructures in not only Si-based materials but also several conventional TE materials such as Bi_2_Te_3_ [27,28], skutterudites [29,30], and SnTe [31], where the reduced *κ*_lat_ and enhanced *zT* have been reported. We confirm that the MS method can produce nanoscale precipitates of various silicides (CrSi_2_ [32], CoSi_2_ [33], TiSi_2_, and VSi_2_ [34]) dispersed homogenously in the Si matrix. Finally, we report on our discovery of the promising new metal silicide for TEs of YbSi_2_, which exhibits a high power factor (*S*^2^*σ*) at room temperature [35,36].

## 2. Bulk Nano-Si Thermoelectric Material

Bulk nanostructured TE materials are traditionally synthesized by grinding the materials into very small particles and consolidating these materials into polycrystalline bulks [19,23]. However, synthesizing fine nanostructured bulk Si via this method is difficult because Si nanopowders are easily oxidized during the grinding process. Therefore, our group has proposed an alternative synthesis of nanostructured bulk Si by precipitating nanoscale particles in the Si matrix [26]. This naturally forms nanostructures and effectively prevents oxidation.

**Figure 1:**
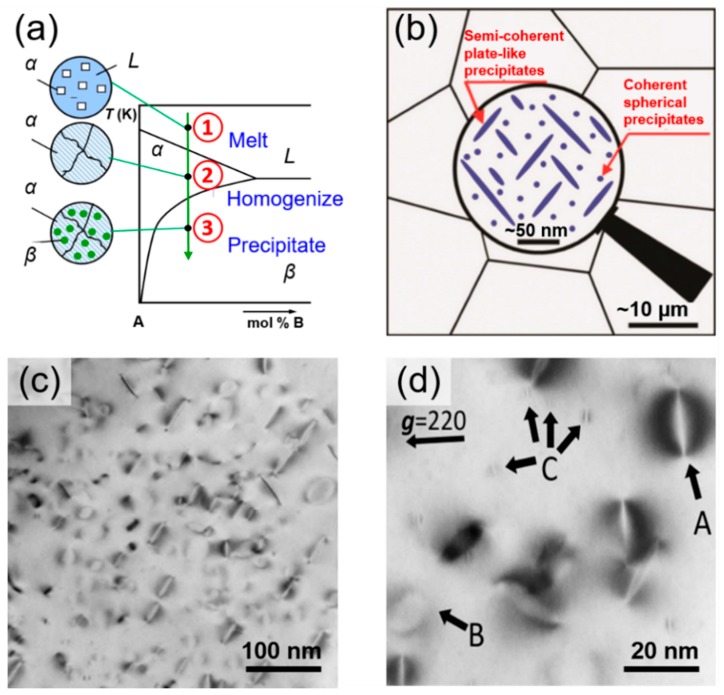
(**a**) Precipitates naturally form when the melt cools from the *α*-phase region in a binary phase diagram. (**b**) Micro- and nanoscale schematics of a nanostructured bulk material containing various types of precipitates. (**c**) Low- and (**d**) high-magnification bright-field transmission electron microscopy (TEM) images of nanostructured bulk Si, showing the homogeneous distribution of nanoscale precipitates in the Si matrix. Contrast shows strain arising from the precipitates. In (**d**), the arrows A and B indicate plate-like precipitates along (high contrast) and perpendicular (lesser contrast) to the electron beam, respectively. The arrow C indicates very small (the average diameter < 5 nm) precipitates with a butterfly-like shade. Reproduced with permission from Ref. [26]. Copyright 2009, the Royal Society of Chemistry.

In the Si–P phase diagram [37], the solubility of P in Si is largely temperature-dependent. At high temperature, a small percentage of P can dissolve into Si; at room temperature, almost no P dissolves. Therefore, when a melt of highly P-doped Si cools from the *α*-phase region (Figure 1a), it is expected that P-rich precipitates form in the Si matrix. By using this method, our group has successfully synthesized nanostructured bulk Si [26]. We confirmed that two types of precipitates form in the Si matrix: semi-coherent plate-shaped precipitates of several dozen nanometers and coherent spherical-shaped precipitates of a few nanometers in size, as shown in Figure 1b–d. Transmission electron microscopy/energy-dispersive X-ray spectroscopy (TEM/EDS) analysis reveals that the precipitates are Si–P binary compounds. The EDS point-analysis has revealed that the precipitates are a Si–P binary compound. However, because the size of the precipitates is too small for the quantitative EDS analysis, the chemical composition, that is, the Si/P ratio has not been determined.

**Figure 2:**
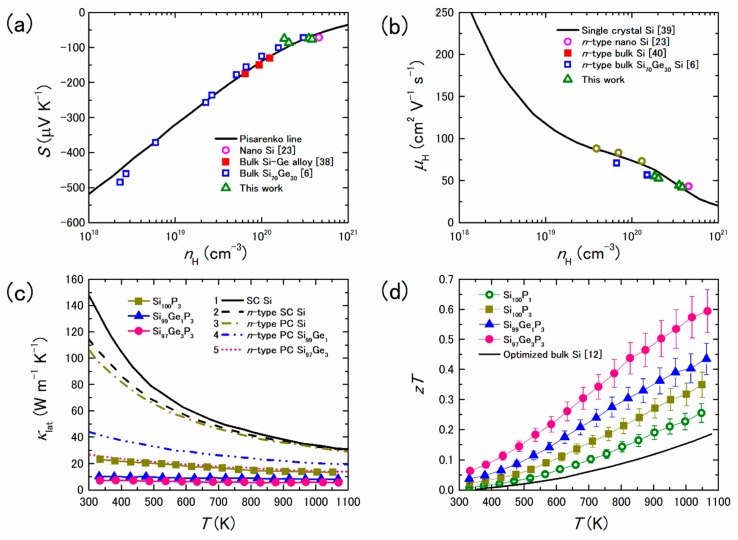
(**a**) Seebeck coefficient *S* and (**b**) Hall carrier mobility *μ*_H_ as a function of Hall carrier concentration *n*_H_ at 300 K for various types of Si. Temperature dependences of (**c**) lattice thermal conductivity *κ*_lat_ and (**d**) dimensionless figure of merit *zT* of nanostructured bulk Si containing small amounts of Ge. Reproduced with permission from Ref. [26]. Copyright 2009, the Royal Society of Chemistry.

To understand the effects of the precipitates on the electrical properties, we added theoretically predicted lines on the *S* vs. *n*_H_ relationship (Figure 2a) and the *µ*_H_ vs. *n*_H_ relationship (Figure 2b), where *n*_H_ and *µ*_H_ are the Hall carrier concentration and the Hall mobility, respectively. The line added on the *S* vs. *n*_H_ relationship is called the Pisarenko line. Moreover, the literature values from References [38,39,40] were added for comparison. As shown in Figure 2a,b, all experimental *S* and *μ*_H_ values of nanostructured bulk Si are on the Pisarenko line and the line obtained by fitting the literature data for non-nanostructured Si, respectively, meaning that the nanoscale precipitates do not significantly affect the electrical properties. However, the *κ*_lat_ of nanostructured bulk Si are much lower than that of single crystalline (SC), heavily doped single crystalline Si (*n*-type SC Si), and heavily doped polycrystalline Si (*n*-type PC Si), as shown in Figure 2c. These low *κ*_lat_ values mainly arise from phonon scattering by the nanoscale precipitates. The maximum *zT* value approaches 0.6 at 1050 K for nanostructured bulk Si containing 3% Ge; this is approximately three times higher than the *zT* value of optimized bulk Si, as shown in Figure 2d.

## 3. Synthesis and Size Control of Si and Metal Silicide Nanocomposites by Melt-Spinning

**Figure 3:**
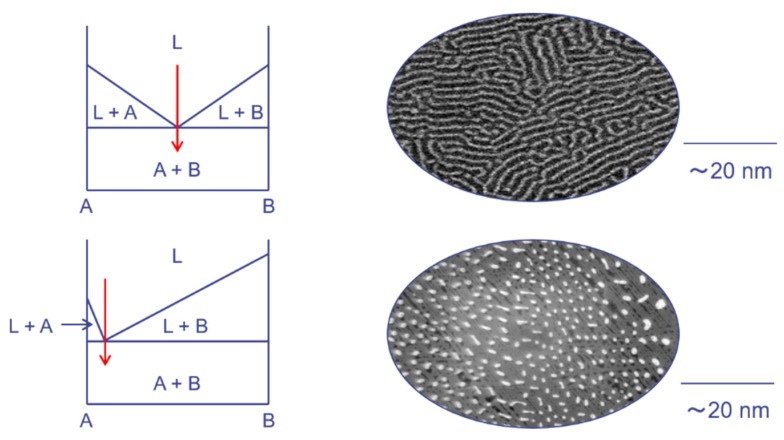
Relationship between phase diagram and nanostructure. According to the position of the eutectic point, nano-lamellar or nano-dot structure can be obtained by melt-spinning (MS).

At the eutectic composition, nanocomposites with homogeneous distributions of secondary phases in the primary-phase matrix can be obtained. Several metal silicides may form such nanoscale precipitates in Si matrices because they have a pseudo-binary eutectic point with Si. As summarized in Figure 3, the structural characteristic of the secondary phase, i.e., nano-lamellar or nano-dot, depends on the position of the eutectic point. Among various metal silicides, CrSi_2_, CoSi_2_, and TiSi_2_ have eutectic points with Si at intermediate dopant concentrations, while VSi_2_ has a eutectic point with Si at low V concentrations.

**Figure 4:**
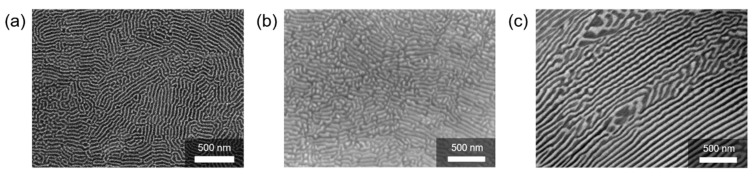
Scanning electron microscopy (SEM) images of nano-lamellar structures obtained by MS at the eutectic points between Si and metal silicides: (**a**) Si–CrSi_2_, (**b**) Si–CoSi_2_, and (**c**) Si–TiSi_2_.

Figure 4 shows scanning electron microscopy (SEM) images of the nano-lamellar structures obtained by MS at the eutectic points between Si and various metal silicides. All composites comprised two phases, i.e., white and dark areas corresponding to the metal silicide and Si matrix, respectively. The TE properties of the Si–CrSi_2_ system (thin ribbons) and the Si–CoSi_2_ system (bulk samples) have been characterized and reported by the authors’ group in reference number [32] and [33], respectively.

**Figure 5:**
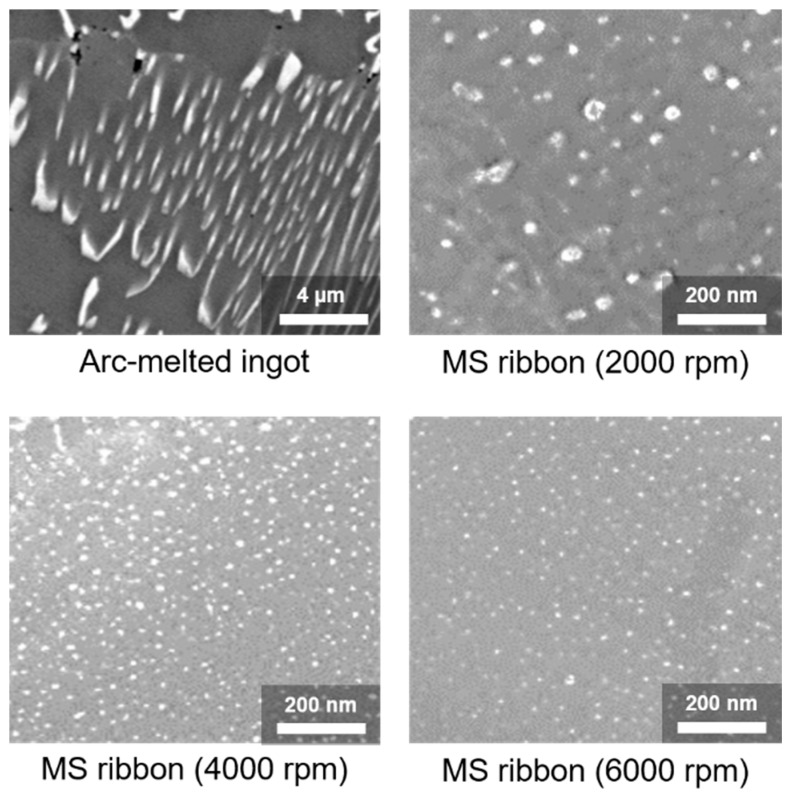
SEM images of nano-dot structures obtained by MS at the eutectic point between Si and VSi_2_, showing that larger cooling rates yield smaller dot sizes. Reproduced with permission from Ref. [34]. Copyright 2016, Springer Nature.

**Figure 6:**
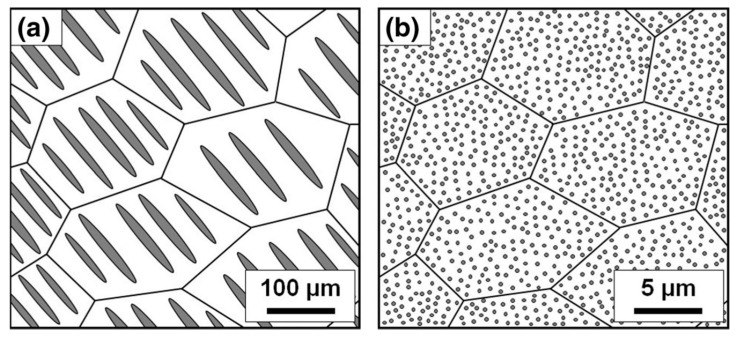
Schematics comparing micro- and nanocomposites synthesized by (**a**) arc melting (normal cooling rate) and (**b**) MS (high cooling rate). Reproduced with permission from Ref. [34]. Copyright 2016, Springer Nature.

As shown in Figure 5, the nano-dot structure was obtained in the Si–VSi_2_ system at the eutectic composition. Because the eutectic point existed at very low V contents (3 at.% V), the nano-dot structure was obtained. Moreover, the dot size was controlled by adjusting the cooling speed, that is, higher cooling rates corresponded to smaller dots, as summarized in Figure 5 and Figure 6. The VSi_2_ nano-precipitates scattered phonons more effectively than charge carriers, thus enhancing *zT* by approximately 40%; it was maximized at 0.23 at 1070 K [34].

## 4. Promising New Metal Silicide for TE Devices

**Figure 7:**
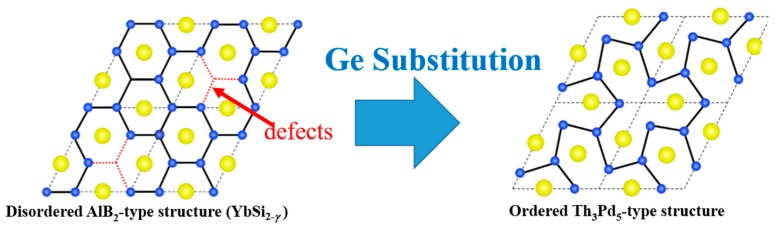
Schematic of crystal structure of YbSiGe projected along the *c* axis, showing a structural transition from the disordered AlB_2_-type structure to the ordered Th_3_Pd_5_-type structure. Yellow and blue balls represent Yb and Si/Ge, respectively. Reproduced with permission from Ref. [36]. Copyright 2018, AIP Publishing.

Our group has discovered that YbSi_2−*δ*_ exhibits a high power factor of 2.2 mW·m^−1^·K^−2^ at room temperature [35]. Because of the mixed valence behavior of Yb, YbSi_2−*δ*_ has an extraordinarily high *S* value despite its metallically high σ. Furthermore, we have demonstrated that the power factor of YbSi_2−*δ*_ can be improved by substituting Si by Ge. The substitution gradually changed the structure from AlB_2_ type with random defects to Th_3_Pd_5_ type with ordered defects, reducing the level of disordered defects, as shown in Figure 7.

**Figure 8:**
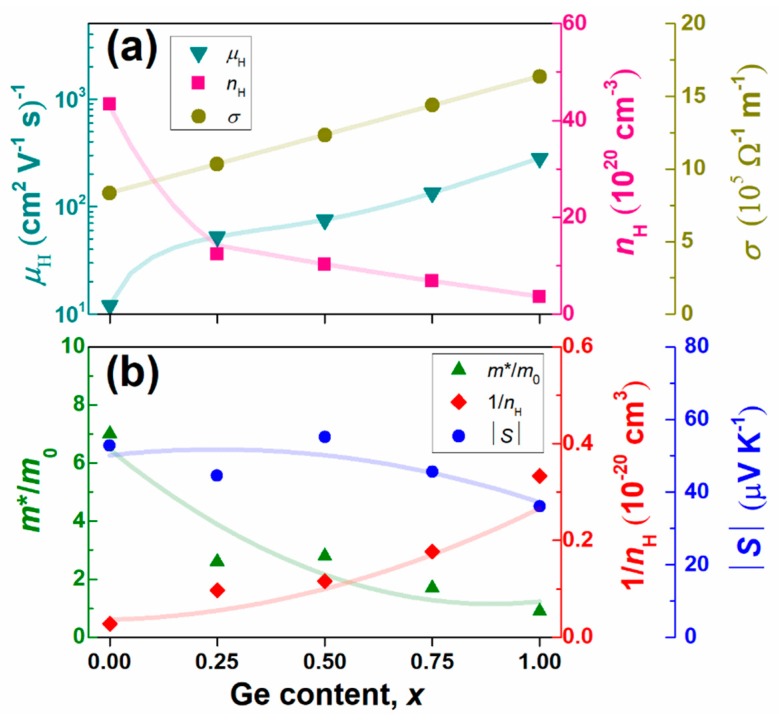
Composition dependences of (**a**) the Hall mobility *µ*_H_, Hall concentration *n*_H_, and electrical conductivity *σ* (=*e*·*µ*_H_·*n*_H_) and (**b**) absolute *S* |*S*|, effective mass m*/*m*^0^, and 1/*n*_H_ for Yb(Si_1−*x*_Ge*_x_*)_2−*δ*_ (0 ≤ *x* ≤ 1.0). All symbols represent experimental data obtained at 300 K. The lines are guides to the eye. Reproduced with permission from Ref. [36]. Copyright 2018, AIP Publishing.

The composition dependences of the Hall mobility *µ*_H_, Hall concentration *n*_H_, and *σ* (=*e*·*µ*_H_·*n*_H_) values are shown in Figure 8a. The *n*_H_ values decreased with the increasing Ge content, possibly because of the reduced density of states near the Fermi level. Moreover, the valence states of Yb in the compounds tended to decrease from 3^+^ to 2^+^ with the increasing Ge content, which may reduce the number of conduction-band electrons, thus reducing *n*_H_. Meanwhile, *µ*_H_ values increased considerably with the increasing Ge content, possibly because of changes in the band structure, reductions in electron scattering, and the removal of disordered defects in the Si sub-lattices via the structural transition discussed above. Based on the results of *n*_H_ and *µ*_H_, the electrical conductivity *σ* (=*e*·*µ*_H_·*n*_H_) was increased approximately linearly with the Ge content.

**Figure 9:**
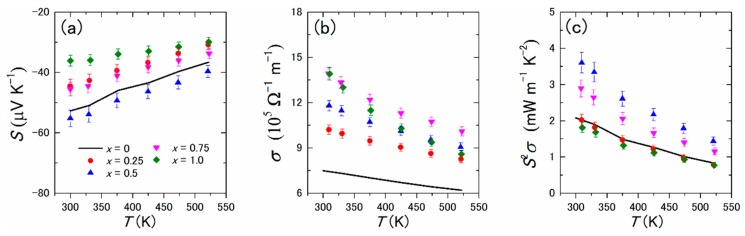
Temperature dependences of (**a**) the Seebeck coefficient *S*, (**b**) electrical conductivity *σ*, and (**c**) power factor *S*^2^*σ* of polycrystalline bulk samples of Yb(Si_1−*x*_Ge*_x_*)_2−*δ*_ (*x* = 0, 0.25, 0.50, 0.75, and 1.0). Reproduced with permission from Ref. [36]. Copyright 2018, AIP Publishing.

Figure 9 shows the electrical properties of polycrystalline bulk samples of Yb(Si_1−*x*_Ge*_x_*)_2−*δ*_ (*x* = 0.25, 0.50, 0.75, and 1.0). The negative values of *S* indicate that electrons were the majority charge carriers; the absolute *S* was maximized at *x* = 0.5. As shown in Figure 8b, the values of m*/*m*_0_ and 1/*n*_H_ show trends opposing those with the Ge content, meaning that an optimized Ge content existed at which the absolute *S* was maximized. The values of *σ* increased with the increasing Ge content, mainly because of the increased *μ*_H_. The maximum *S*^2^*σ* value was 3.6 mW·m^−1^·K^−2^ at room temperature at *x* = 0.5; this value is comparable to or higher than those of conventional TE materials such as Bi_2_Te_3_.

**Figure 10:**
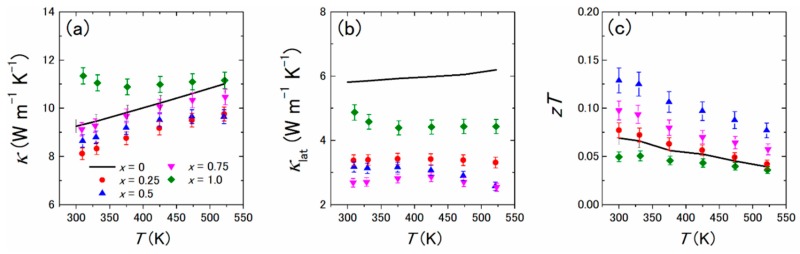
Temperature dependences of (**a**) total thermal conductivity *κ*, (**b**) lattice thermal conductivity *κ*_lat_, and (**c**) dimensionless figure of merit *zT* for polycrystalline bulk samples of Yb(Si_1−*x*_Ge*_x_*)_2−*δ*_ (*x* = 0, 0.25, 0.50, 0.75, and 1.0). Reproduced with permission from Ref. [36]. Copyright 2018, AIP Publishing.

Figure 10a–c show the temperature dependences of *κ*, *κ*_lat_, and *zT* of Yb(Si_1−*x*_Ge*_x_*)_2−*δ*_ (*x* = 0–1.0). As can be seen in Figure 10b, the samples with *x* = 0.25, 0.50, and 0.75 had lower *κ*_lat_ values than those with *x* = 0 and 1, mainly due to the phonon scattering of Si/Ge substitution. The enhanced power factor and reduced *κ*_lat_ led to an improvement of *zT* for *x* = 0.25, 0.50, and 0.75, as can be seen in Figure 10c. The maximum *zT* value was 0.13 at room temperature obtained for the sample with *x* = 0.5.

## 5. Summary

In this review, recent research toward the development of high-efficiency eco-friendly Si-based TE materials was summarized. When a melt of highly P-doped Si is cooled from the *α*-phase, Si–P binary precipitates form naturally in the Si matrix, yielding nanostructured bulk Si. The nanoscale precipitates reduce *κ*_lat_ without significantly affecting the electron transport properties, thus enhancing *zT*. In addition to this natural nanostructuring procedure, our group has proposed the rapid-solidification MS method to synthesize Si–metal silicide nanocomposites. At the eutectic composition of Si and a given metal silicide, a eutectic structure with a homogeneous dispersion of metal silicides in the Si matrix can be obtained. Furthermore, with varied cooling rates, the eutectic structure feature size can be controlled, that is, higher cooling rates correspond to smaller sizes, as demonstrated in the Si–VSi_2_ system. Finally, we have discovered a promising high-power-factor TE metal silicide, YbSi_2_. Because of the mixed valence state of Yb, this metal silicide exhibits a large *S* with a metallically high *σ*, yielding a high room-temperature power factor. Furthermore, the power factor can be enhanced by the substitution of Ge for Si.

## References

[1] Bell L.E. (2008). Cooling, Heating, Generating Power, and Recovering Waste Heat with Thermoelectric Systems. Science.

[2] Snyder G.J., Toberer E.S. (2008). Complex Thermoelectric Materials. Nat. Mater..

[3] Venkatasubramanian R., Siivola E., Colpitts T., O’Quinn B. (2001). Thin-Film Thermoelectric Devices with High Room-Temperature Figures of Merit. Nature.

[4] Heremans J.P., Jovovic V., Toberer E.S., Saramat A., Kurosaki K., Charoenphakdee A., Yamanaka S., Snyder G.J. (2008). Enhancement of Thermoelectric Efficiency in PbTe by Distortion of the Electronic Density of States. Science.

[5] Steele M.C., Rosi F.D. (1958). Thermal Conductivity and Thermoelectric Power of Germanium Silicon Alloys. J. App. Phys..

[6] Dismukes J.P., Ekstrom L., Steigmeier E.F., Kudman I., Beers D.S. (1964). Thermal and Electrical Properties of Heavily Doped Ge-Si Alloys up to 1300 K. J. Appl. Phys..

[7] Zaitsev V.K., Fedorov M.I., Gurieva E.A., Eremin I.S., Konstantinov P.P., Samunin A.Y., Vedernikov M.V. (2006). Highly Effective Mg2Si_1−x_Sn_x_ Thermoelectrics. Phys. Rev. B.

[8] Liu W., Tan X., Yin K., Liu H., Tang X., Shi J., Zhang Q., Uher C. (2012). Convergence of Conduction Bands as a Means of Enhancing Thermoelectric Performance of *n*-Type Mg_2_Si_1-x_Sn_x_ Solid Solutions. Phys. Rev. Lett..

[9] Zaitsev V.K., Rowe D.M. (1995). Thermoelectrics Handbook.

[10] Fedorov M.I., Zaitsev V.K., Rowe D.M. (2006). Thermoelectrics Handbook.

[11] Weber L., Gmelin E. (1991). Transport Properties of Silicon. Appl. Phys. A.

[12] Zhu G.H., Lee H., Lan Y., Wang X., Joshi G., Wang D., Yang J., Vashaee D., Guilbert H., Pillitteri A. (2009). Increased Phonon Scattering by Nanograins and Point Defects in Nanostructured Silicon with a Low Concentration of Germanium. Phys. Rev. Lett..

[13] Tritt T.M. (1999). Holey and Unholey Semiconductors. Science.

[14] Xie H.H., Wang H., Pei Y.Z., Fu C.G., Liu X.H., Snyder G.J., Zhao X.B., Zhu T.J. (2013). Beneficial Contribution of Alloy Disorder to Electron and Phonon Transport in Half-Heusler Thermoelectric Materials. Adv. Funct. Mater..

[15] Sales B.C., Mandrus D., Williams R.K. (1996). Filled Skutterudite Antimonides: A New Class of Thermoelectric Materials. Science.

[16] Shi X., Yang J., Salvador J.R., Chi M., Cho J.Y., Wang H., Bai S., Yang J., Zhang W., Chen L. (2011). Multiple-Filled Skutterudites: High Thermoelectric Figure of Merit through Separately Optimizing Electrical and Thermal Transports. J. Am. Chem. Soc..

[17] Nielsen M.D., Ozolins V., Heremans J.P. (2013). Lone Pair Electrons Minimize Lattice Thermal Conductivity. Energy Environ. Sci..

[18] Zhao L.D., Lo S.H., Zhang Y., Sun H., Tan G., Uher C., Wolverton C., Dravid V.P., Kanatzidis M.G. (2014). Ultralow Thermal Conductivity and High Thermoelectric Figure of Merit in SnSe Crystals. Nature.

[19] Poudel B., Hao Q., Ma Y., Lan Y., Minnich A., Yu B., Yan X., Wang D., Muto A., Vashaee D. (2008). High-Thermoelectric Performance of Nanostructured Bismuth Antimony Telluride Bulk Alloys. Science.

[20] Biswas K., He J., Blum I.D., Wu C.I., Hogan T.P., Seidman D.N., Dravid V.P., Kanatzidis M.G. (2012). High-Performance Bulk Thermoelectrics with All-Scale Hierarchical Architectures. Nature.

[21] Hochbaum A.I., Chen R., Delgado R.D., Liang W., Garnett E.C., Najarian M., Majumdar A., Yang P. (2008). Enhanced Thermoelectric Performance of Rough Silicon Nanowires. Nature.

[22] Boukai A.I., Bunimovich Y., Tahir-Kheli J., Yu J.K., Goddard W.A., Heath J.R. (2008). Silicon Nanowires as Efficient Thermoelectric Materials. Nature.

[23] Bux S.K., Blair R.G., Gogna P.K., Lee H., Chen G., Dresselhaus M.S., Kaner R.B., Fleurial J.P. (2009). Nanostructured Bulk Silicon as an Effective Thermoelectric Material. Adv. Funct. Mater..

[24] Dresselhaus M.S., Chen G., Tang M.Y., Yang R., Lee H., Wang D., Ren Z., Fleurial J.P., Gogna P. (2007). New Directions for Low-Dimensional Thermoelectric Materials. Adv. Mater..

[25] Kurosaki K., Yusufu A., Miyazaki Y., Ohishi Y., Muta H., Yamanaka S. (2016). Enhanced Thermoelectric Properties of Silicon via Nanostructuring. Mater. Trans..

[26] Yusufu A., Kurosaki K., Miyazaki Y., Ishimaru M., Kosuga A., Ohishi Y., Muta H., Yamanaka S. (2014). Bottom-Up Nanostructured Bulk Silicon: A Practical High-Efficiency Thermoelectric Material. Nanoscale.

[27] Xie W., He J., Kang H.J., Tang X., Zhu S., Laver M., Wang S., Copley J.R.D., Brown C.M., Zhang Q. (2010). Identifying the Specific Nanostructures Responsible for the High Thermoelectric Performance of (Bi,Sb)_2_Te_3_ Nanocomposites. Nano Lett..

[28] Tang X., Xie W., Li H., Zhao W., Zhang Q., Niino M. (2007). Preparation and Thermoelectric Transport Properties of High-Performance *p*-type Bi_2_Te_3_ with Layered Nanostructure. Appl. Phys. Lett..

[29] Li H., Tang X., Su X., Zhang Q. (2008). Preparation and Thermoelectric Properties of High-Performance Sb Additional Yb_0.2_Co_4_Sb_12+y_ Bulk Materials with Nanostructure. Appl. Phys. Lett..

[30] Li H., Tang X., Zhang Q., Uher C. (2008). Rapid Preparation Method of Bulk Nanostructured Yb_0.3_Co_4_Sb_12+y_ Compounds and Their Improved Thermoelectric Performance. Appl. Phys. Lett..

[31] Tan L.P., Sun T., Fan S., Ramanujan R.V., Hng H.H. (2014). Facile Precipitation of Two Phase Alloys in SnTe_0.75_Se_0.25_ with Improved Power Factor. J. Alloys Compd..

[32] Norizan M.N., Miyazaki Y., Ohishi Y., Muta H., Kurosaki K., Yamanaka S. (2018). The Nanometer-Sized Eutectic Structure of Si/CrSi_2_ Thermoelectric Materials Fabricated by Rapid Solidification. J. Electron. Mater..

[33] Xie J., Ohishi Y., Ichikawa S., Muta H., Kurosaki K., Yamanaka S. (2017). Thermoelectric Properties of Si/CoSi_2_ Sub-Micrometer Composites Prepared by Melt-Spinning Technique. J. Appl. Phys..

[34] Tanusilp S., Kurosaki K., Yusufu A., Ohishi Y., Muta H., Yamanaka S. (2017). Enhancement of Thermoelectric Properties of Bulk Si by Dispersing Size-Controlled VSi_2_. J. Electron. Mater..

[35] Tanusilp S., Ohishi Y., Muta H., Yamanaka S., Nishide A., Hayakawa J., Kurosaki K. (2018). Ytterbium Silicide (YbSi_2_): A Promising Thermoelectric Material with a High Power Factor at Room Temperature. Phys. Status Solidi RRL.

[36] Tanusilp S., Ohishi Y., Muta H., Nishide A., Hayakawa J., Kurosaki K. (2018). High Thermoelectric Power Factor of Ytterbium Silicon-Germanium. Appl. Phys. Lett..

[37] Olesinski R.W., Kanani N., Abbaschian G.J. (1985). The P-Si (Phosphorus-Silicon) System. Bull. Alloy Phase Diagr..

[38] Golikova O.A., Iordanishvili E.K., Petrov A.V., Tela F.T. (1966). Electrical properties of solid solutions in the Si-Ge system (Electric conductivity and thermal emf of solid solutions of silicon-germanium with near silicon composition and various current-carrier concentrations and test temperatures). Sov. Phys. Solid State.

[39] Masetti G., Severi M., Solmi S. (1983). Modeling of Carrier Mobility against Carrier Concentration in Arsenic-, Phosphorus-, and Boron-Doped Silicon. IEEE Trans. Electron Devices.

[40] Morin F., Maita J. (1954). Electrical Properties of Silicon Containing Arsenic and Boron. Phys. Rev..

